# Diuretic effect of co-administration of furosemide and albumin in comparison to furosemide therapy alone: An updated systematic review and meta-analysis

**DOI:** 10.1371/journal.pone.0260312

**Published:** 2021-12-01

**Authors:** Tao Han Lee, George Kuo, Chih-Hsiang Chang, Yen Ta Huang, Chieh Li Yen, Cheng-Chia Lee, Pei Chun Fan, Jia-Jin Chen

**Affiliations:** 1 Department of Nephrology, Kidney Research Center, Chang Gung Memorial Hospital, Taoyuan, Taiwan; 2 Graduate Institute of Clinical Medical Science, College of Medicine, Chang Gung University, Taoyuan, Taiwan; 3 Department of Surgery, National Cheng Kung University Hospital, College of Medicine, National Cheng Kung University, Tainan, Taiwan; Universita degli Studi di Perugia, ITALY

## Abstract

**Background:**

It has been a matter of much debate whether the co-administration of furosemide and albumin can achieve better diuresis and natriuresis than furosemide treatment alone. There is inconsistency in published trials regarding the effect of this combination therapy. We, therefore, conducted this meta-analysis to explore the efficacy of furosemide and albumin co-administration and the factors potentially influencing the diuretic effect of such co-administration.

**Methods:**

In accordance with the Preferred Reporting Items for Systematic Reviews and Meta-Analyses (PRISMA) guidelines, we searched the PubMed, Embase, Medline, and Cochrane databases. Prospective studies with adult populations which comparing the effect of furosemide and albumin co-administration with furosemide alone were included. The outcomes including diuretic effect and natriuresis effect measured by hourly urine output and hourly urine sodium excretion from both groups were extracted. Random effect model was applied for conducting meta-analysis. Subgroup analysis and sensitivity analysis were performed to explore potential sources of heterogeneity of treatment effects.

**Results:**

By including 13 studies with 422 participants, the meta-analysis revealed that furosemide with albumin co-administration increased urine output by 31.45 ml/hour and increased urine excretion by 1.76 mEq/hour in comparison to furosemide treatment alone. The diuretic effect of albumin and furosemide co-administration was better in participants with low baseline serum albumin levels (< 2.5 g/dL) and high prescribed albumin infusion doses (> 30 g), and the effect was more significant within 12 hours after administration. Diuretic effect of co-administration was better in those with baseline Cr > 1.2 mg/dL and natriuresis effect of co-administration was better in those with baseline eGFR < 60 ml/min/1.73m2.

**Conclusion:**

Co-administration of furosemide with albumin might enhance diuresis and natriuresis effects than furosemide treatment alone but with high heterogeneity in treatment response. According to the present meta-analysis, combination therapy might provide advantages compared to the furosemide therapy alone in patients with baseline albumin levels lower than 2.5 g/dL or in patients receiving higher albumin infusion doses or in patients with impaired renal function. Owing to high heterogeneity and limited enrolled participants, further parallel randomized controlled trials are warranted to examine our outcome.

**Registration:**

PROSEPRO ID: CRD42020211002; https://clinicaltrials.gov/.

## Introduction

The loop diuretic furosemide is commonly used for the management of fluid overload. Although it shows high potency in terms of free water clearance and natriuresis, diuretic resistance is still inevitable in situations complicated by decreased kidney function, hypoalbuminemia, use of nonsteroidal anti-inflammatory drugs (NSAIDs), or congestive heart failure [1–3]. Furosemide is a highly protein-bound organic acid, and that more than 95 percent of furosemide in plasma is bound to albumin. This protein-bound fraction of furosemide reaches the anion transporters at the proximal tubule epithelial cells via blood circulation and then it is translocated into their action sites, the tubule lumen of the ascending limbs of Henle’s loop [4]. However, although physiological theory and the results of animal models consistently support the conclusion that furosemide and albumin co-administration could increase the secretion of active form of furosemide in the renal tubular lumen [1–3,5,6], the evidence from clinical trials supporting such co-administration has remained ambiguous. In 1987, Inoue and his colleagues had first proposed that hypoalbuminemia influences the potency of furosemide and that the co-administration of albumin and furosemide could increase the diuretic response in comparison to furosemide alone in both animal and human models [5]. According to the animal study by Pichette et al., the serum albumin level affects the renal metabolic clearance of furosemide. Hypoalbuminemia, which can in turn result in reduced levels of the active form of furosemide in tubular secretions [6]. Although hypoalbuminemic individuals who were resistant to diuretics also achieved diuresis through the co-administration of furosemide and albumin in the aforementioned studies, other clinical studies and previously published meta-analyses were unable to confirm that phenomenon [7–12]. Because furosemide and albumin constitute a common combination therapy in clinical practice, the inconclusive evidence supporting it, its high cost, and the anaphylactic risk of albumin have motivated us to seek stronger evidence to support its use.

Therefore, in the present study, we investigated the efficacy of furosemide and albumin co-administration through an updated meta-analysis, including an exploration of factors that might interfere with the diuretic effect of furosemide and albumin co-administration.

## Methods

### Literature searches and data sources

Online literature searches of the PubMed, EMBASE, Medline, and Cochrane databases were performed. This database search was last updated on October 18, 2020. The search strategy targeted published clinical trials, including conference abstracts that compared the diuretic effects of furosemide with albumin and furosemide alone in adult populations. The PubMed and EMBASE databases were searched using the terms “furosemide” OR “Lasix”(brand name for furosemide) OR “diuretics” AND “hypoalbuminemia” OR “albumin” limited to the criteria “clinical trials” and “human”. The detailed results of that search process are provided in **S1 Table**. The Medline database was searched using the terms “furosemide” OR “loop diuretics” AND “hypoalbuminemia” OR “albumin” limited to “all adults”. No language restrictions were applied.

### Study selection

Two investigators (T.H. Lee and J.J. Chen) independently evaluated the titles and abstracts of the retrieved studies, and articles were excluded upon initial screening if their titles or abstracts indicated that they were clearly irrelevant to the objective of the current study. Full-text reviews of the articles deemed potentially relevant were then performed to assess their eligibility for inclusion in the meta-analysis. For inclusion in the systematic review, a study had to meet the following criteria: (i) have a study population consisting of adults and have a prospective design, (ii) allocate patients to furosemide or furosemide with albumin treatment groups, and (iii) report one of the following outcomes: urine output rate or cumulative urine amount, urine sodium excretion rate, or cumulative sodium excretion amount. Any disagreement regarding the eligibility of the full-text articles was resolved by consensus. We have registered our study design and protocol in PROSPERO, the present study was approved by the editorial team of PROSPERO: CRD42020211002.

### Data extraction and quality assessment

Data extracted from each of the included studies included the publication year, study design, index disease, sample size, mean age of participants, baseline serum albumin and creatinine levels (if no baseline creatinine levels were reported, the estimated glomerular filtration rate was recorded), pharmacist intervention (furosemide and albumin dosage), outcome of interest (urine output rate or urine sodium excretion rate), and exclusion and inclusion criteria, all of which were independently extracted by two investigators. The study quality of any randomized control trials was assessed using the Revised Cochrane Risk-of-Bias tool for randomized trials (RoB 2) [13,14], an assessment tool developed by the Cochrane Collaboration. A bias assessment of crossover trials was also conducted according to the Cochrane Handbook for Systematic Reviews of Interventions with a modified RoB 2 [14]. To assess the confidence levels for each outcome effect estimate, evidence quality was rated as high risk, some concerns, or low risk. Disagreements among two investigators (T.H. Lee and J.J. Chen) were resolved by consensus with another author (G.K.).

### Outcome measures

Outcomes of interest were urinary output and urinary sodium excretion of furosemide combined with albumin in comparison with furosemide alone. Most of the included studies reported cumulative urine output at 6 hours, 8 hours, or 24 hours. Two studies reported urine output rates in milliliters per minute (ml/min). Similarly, urine sodium excretion levels were reported in different ways in the included studies as cumulative urinary sodium excretion amount within 4 hours, 6 hours, 8 hours, or 24 hours. Therefore, we analyzed urine output and urine sodium excretion as urine output rate (ml/hour) and urinary sodium excretion rate (mEq/hour). For these two continuous outcomes, the mean with standard deviation of the hourly urine output or hourly sodium excretion was extracted and calculated from the included studies.

### Statistical analysis

In this meta-analysis, the differences in urine output and urinary sodium excretion between the intervention and control groups were defined as the primary outcome measures. For parallel design trials, the mean difference, standard deviations (SD) of the mean difference, and standard error of mean difference were calculated from the reported outcomes of the intervention and control groups. For crossover trials, we assumed that there were no carry-over and period effects. The treatment effect was defined as within-individual mean difference between the intervention and control management for crossover trials. Owing to neither within-individual difference and standard deviation of within-individual difference nor the standard error for the within-individual differences being reported in the included crossover trials, we then imputed a correlation coefficient of 0.5 to obtain the standard error of within-individual mean difference [14–17]. The data from individual studies were pooled using the random effect model. Outcomes from parallel and crossover studies were extracted and analyzed using the generic inverse variance method (*metagen* function in the *meta* package) [18]. Heterogeneity was examined via *I*^*2*^ index, with *I*^*2*^ < 25%, 25–50%, and > 50% indicating mild, moderate, and high heterogeneity, respectively. Sensitivity analyses were performed to assess the robustness of results. Each sensitivity analysis was performed by excluding studies with outlier results, studies with high risk of bias, or studies with crossover designs. To explore possible sources of treatment effect heterogeneity, subgroup analyses were performed by examining whether different treatment effects existed across the following variables: (1) different index diseases, (2) exclusion or inclusion of AKI patients, (3) baseline serum creatinine level (> 1.2 mg/dL or ≤ 1.2 mg/dL), (4) baseline serum albumin level (≥ 2.5 g/dL or < 2.5 g/dL), (5) prescribed intravenous furosemide dose (≥ 60 mg or < 60 mg), (6) prescribed albumin dose (≥ 30 g or < 30 g), (7) duration of observation (≤ 12 hours or > 12hours), and (8) baseline eGFR (≥ 60 ml/min/1.73m2 or < 60 ml/min/1.73m2). The differences in treatment effect were tested between subgroups, and a p-value < 0.1 indicated a potential subgroup effect [18,19]. Three studies reported dose of albumin based on body weight; therefore, we assumed baseline body weight 60 kg and considered the prescribed albumin dose at least 30g in these three studies (Akcicek, 1995; Ghafari, 2011; Sjöström, 1989) [9,20,21]. Publication bias was assessed using funnel plots. In general, a two-sided P value < 0.05 was considered statistically significant. Risk of bias plots were created by using an online resource: Risk-of-bias VISualization [19,22]. This meta-analysis was conducted with R version 4.0.3 (2020-10-10) [23]. The quality of evidence for the treatment effect of albumin and furosemide co-administration in this meta-analysis was assessed based on the guidelines of the GRADE Working Group methodology [24]. We summarized the results in a table, which was constructed using the online GRADE Profiler (**S2 Table**) [24].

## Results

### Search results and study characteristics

The study selection process is shown in **S1 Fig**. The comprehensive search identified 156 potentially eligible studies from the PubMed database, 123 potentially eligible studies from the EMBASE database,132 potentially eligible studies from the Medline database, and 1 potentially eligible study from the Cochrane database. After screening the titles and abstracts of these potentially eligible studies, 23 full-text articles were further assessed for eligibility. After excluding 10 studies for having non-adult populations, duplicate cohorts, and different outcomes of interest, 13 studies were ultimately included in our analysis. Review articles and meta-analyses were not included in our analysis, but their references were screened and searched for relevant studies. The details of the search strategy, search results, and reasons for study exclusion are summarized in **S1 Fig and S3 Table.**

The included trials were published between 1987 and 2020, with crossover designs used in 9 of the studies and parallel-group designs used in 4 of the studies. Combining all the studies, data for a total of 422 individuals were analyzed. The population studied included patients with nephrotic syndrome, liver cirrhosis, hypoalbuminemia of unspecified cause and critically ill (ICU) patients. The majority of studies reported at least one of the following forms of data: urine output rate or urine output volume, urinary sodium excretion rate, or cumulative excretory sodium amount. **Table 1** presents the basic characteristics of the included study patients, and **Table 2** presents the outcomes of the 13 included studies.

**Table 1:** Basic characteristics and study design of enrolled studies.

Study	Study design	Index disease	Mean age	Sample size	Average or range of baseline albumin(g/dL)	Average or range of baseline creatinine(mg/dL)	Exclusion of AKI	Dose of furosemide (mg)	Dose of albumin (g)
Akcicek, 1995 [9]	Randomized, crossover	nephrotic syndrome	NR	8	1.1 to 1.2	1.20 to 2.40	N	60mg bolus than 40mg/hr for 4 hrs	0.5g/kg
Chalasani, 2001 [25] (premix)	Randomized, crossover	liver cirrhosis,	51.2	13	3.0	0.99	N	40mg	25
Chalasani, 2001 (separate)			51.2	13	3.0	0.99	N	40mg	25
Fliser, 1999 [26]	Randomized, crossover	nephrotic syndrome	48	9	NR	NR*	N	60mg	40
Ghafari, 2011 [20]	Randomized, crossover	nephrotic syndrome	NR	10	NR	NR	N	2mg/kg	0.5g/kg
Gentilini, 1999 [27] (protocol 1)	Randomized, parallel	liver cirrhosis	62.2	126	3.12	0.975	N	25mg to 160mg/day	12.5g
Hsu, 2006 [28] (CCr ≤20)	Randomized, crossover	critical illness	71	21	2.43	3.75	Y	60mg	40
Hsu, 2006 (CCr >20)			69	21	2.27	1.36	Y	60mg	40
Inoue, 1987 [5]	Randomized, crossover	hypoalbuminemia	64.6	16	2.2	NR	N	20 to 60mg	6^Ø^
Mahmoodpoor, 2020 [10]	Randomized, parallel	critical illness	71.1	38	2.6	NR^†^	N	20mg	20
Na, 2001 [29]	Randomized, crossover	nephrotic syndrome	41.1	7	1.7	1.59	N	160mg	25
Nakamura, 2013 [30]	Randomized, parallel	liver cirrhosis	61.3	66	2.61	0.84	N	20 mg/day, for 5 days	10
Phakdeekitcharoen, 2012 [7]	Randomized, crossover	hypoalbuminemia	66.4	24	2.98	2.18	Y	40mg	25
Simon, 2018 [31]	Randomized, parallel	critical illness	63.1	45	2.00	0.67	Y	NR^±^	40
Sjöström, 1989 [21]	Randomized, crossover	nephrotic syndrome	48	5	2.7	NR^‡^	N	40mg	0.5g/kg

CCr: Creatinine clearance rate.

*: No baseline creatinine was reported in this study, but average baseline GFR was 105 ml/min/1.73m2.

†: No baseline creatinine was reported in this study, but average baseline Clcr was 73.2 ml/min.

‡: No baseline creatinine was reported in this study, but average baseline GFR was 78 ml/min/1.73m2.

±: Furosemide dose decided by clinical team.

Ø: Albumin dose was equimolar to furosemide.

**Table 2:** Treatment effect of of intervention and controlled group from enrolled studies.

Study	Study design	Urine output rate (ml/hr) ± SD of furosemide and albumin	Urine output rate (ml/hr) ± SD of furosemide alone	Urine Na excretion rate (meq/hr) ± SD of furosemide and albumin	Urine Na excretion rate (meq/hr) ± SD of furosemide alone	Duration of urine output collection
Akcicek, 1995 [9]	Crossover	82.8 ± 30	46.2 ±15.6	NR	NR	18 hours
Chalasani, 2001 [25] (premix)	Crossover	471.6 ±38.5	447.7 ±38.3	27.5 ±2.3	25.7 ±2.3	6 hours
Chalasani, 2001 (separate)		492.7 ±38.5	447.7 ± 38.3	28.7 ±2.5	25.7 ±2.3	6 hours
Fliser, 1999 [26]	Crossover	178.3 ±7.7	157.4 ±6.6	15.6 ±1.4	12.7 ±1.4	8 hours
Ghafari, 2011 [20]	Crossover	90.6 ±40.5	71.1 ±31.1	10.9 ±0.4	8.7 ±0.2	24 hours
Gentilini, 1999 [27] (protocol 1)	Parallel	NR	NR	4 ±1.2	3.5 ±1.5	24 hours
Hsu, 2006 [28] (CCr ≤20)	Crossover	110 ±56.4	50.5 ±23.9	8.5 ±6.9	3 ±1.4	8 hours
Hsu, 2006 (CCr >20)		293.3 ±139.1	73.9 ±29.9	29.3 ±20.8	4.1 ±2.6	8 hours
Inoue, 1987 [5]	Crossover	183 ±132.6	88.2 ±94.8	NR	NR	NR
Mahmoodpoor, 2020 [10]	Parallel	299.5 ±124	259.1 ±105.5	38.2 ±4.6	38.8 ±6.3	4 hours
Na, 2001 [29]	Crossover	84.5 ±8.3	72.1 ±8.3	5.1 ±1.6	5.4 ±1.2	24 hours
Nakamura, 2013 [30]	Parallel	67.1 ±33.2	58 ±23.7	4.5 ±2.1	4.2 ±2.2	24 hours
Phakdeekitcharoen, 2012 [7]	Crossover	102.9 ±25	101.7 ±30.8	6.4 ± 1.8	6.1 2.5	6 hours
Simon, 2018 [31]	Parallel	136.7 ±65	147.9 ±53.3	NR	NR	24 hours
Sjöström, 1989 [21]	Crossover	450 ±210	420 ±252	NR	NR	4–8 hours

SD: Standard deviation.

### Risk of bias

The results of the estimated risk of bias of the included trials based on the RoB 2 tool developed by the Cochrane Collaboration are summarized in **S2 and S3 Figs**. Some sources of potential bias included the following: (i) eight of the included trials had high risk of bias related to domain 1 and (ii) three of the included studies had high risk of bias related to domain 3, including two studies (Nakamura, 2013; Mahmoodpoor,2020) that reported missing outcome data due to incomplete participant data but did not provide the relevant details and one study (Akcicek,1995) that excluded 4 of 12 participants because of factors that might have influenced the outcome data [9,10,30]. The outcome measurement methods used in all of the included trials had no diagnostic detection bias, and there were no studies that had multiple eligible outcome measurements within the outcome domain, yielding the low risk in measurement outcomes and results reported. Overall, four of the included studies were considered to have high risk of bias (Akcicek, 1995; Inoue, 1987; Nakamura, 2013; Mahmoodpoor, 2020) [5,9,10,30]. Publication bias was assessed by funnel plot and no significant asymmetry was detected (**S4 & S5 Figs**)

### Effect on urine output of furosemide and albumin in comparison to furosemide alone

**Fig 1:**
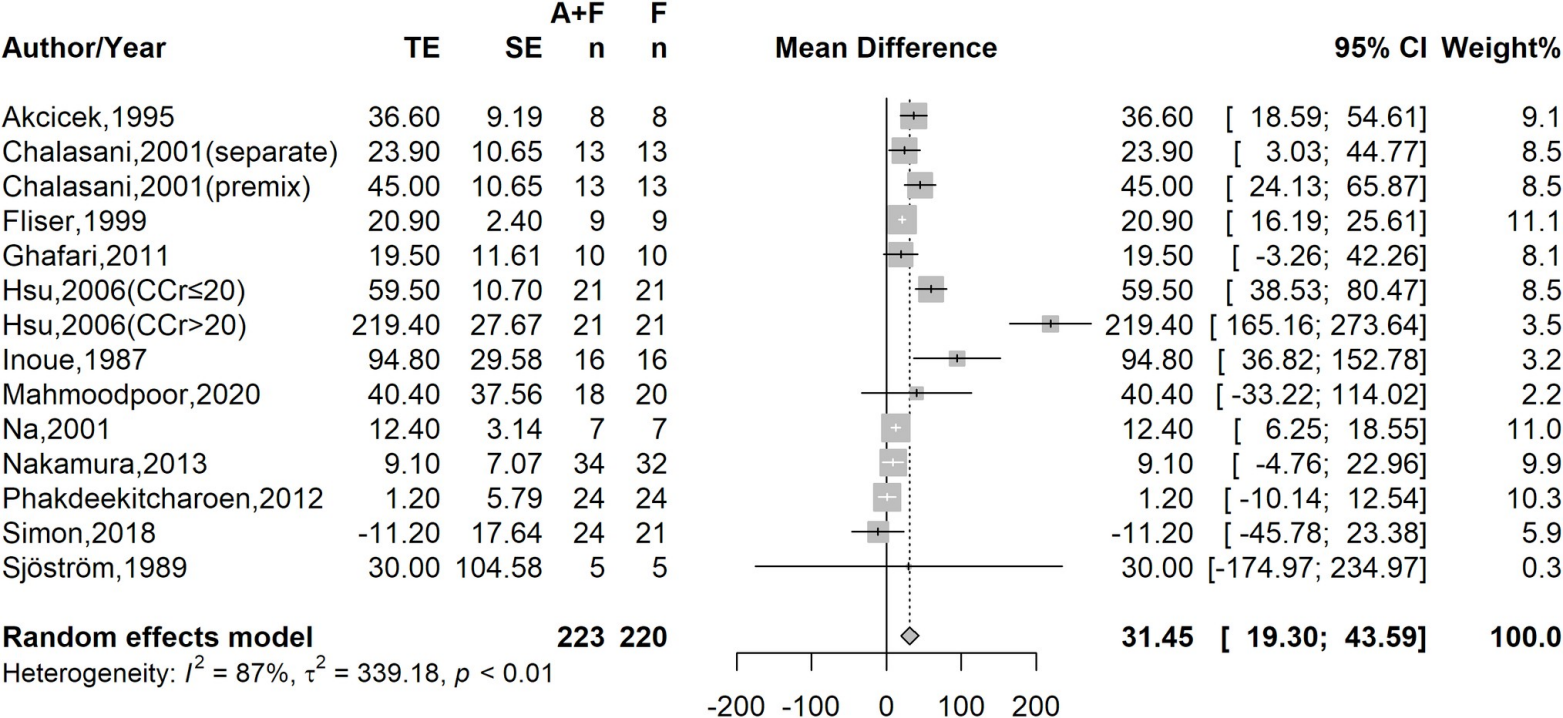
Treatment effect of co-administration furosemide with albumin on urine output rate.

Among the 13 included studies, 5 studies reported the urine output amount within 6 to 8 hours after the administration of furosemide alone or the co-administration of furosemide with albumin, 5 studies reported the urine output amount within 24 hours, and 3 studies reported the urine output rate after treatment. Considering the different methods of reporting urine output among the studies, we unified the reported urine output amounts with different time interval into hourly urine output rates in order to undertake further analysis. The meta-analysis showed that the co-administration of furosemide with albumin increased the mean urine output rate to 31.45 ml/hour (95% CI, 19.30–43.59) above that with furosemide treatment alone (**Fig 1**). However, high heterogeneity across studies (*I*^*2*^ = 87%, p<0.01) was detected.

### Effect on urinary sodium excretion of furosemide and albumin in comparison with furosemide alone

**Fig 2:**
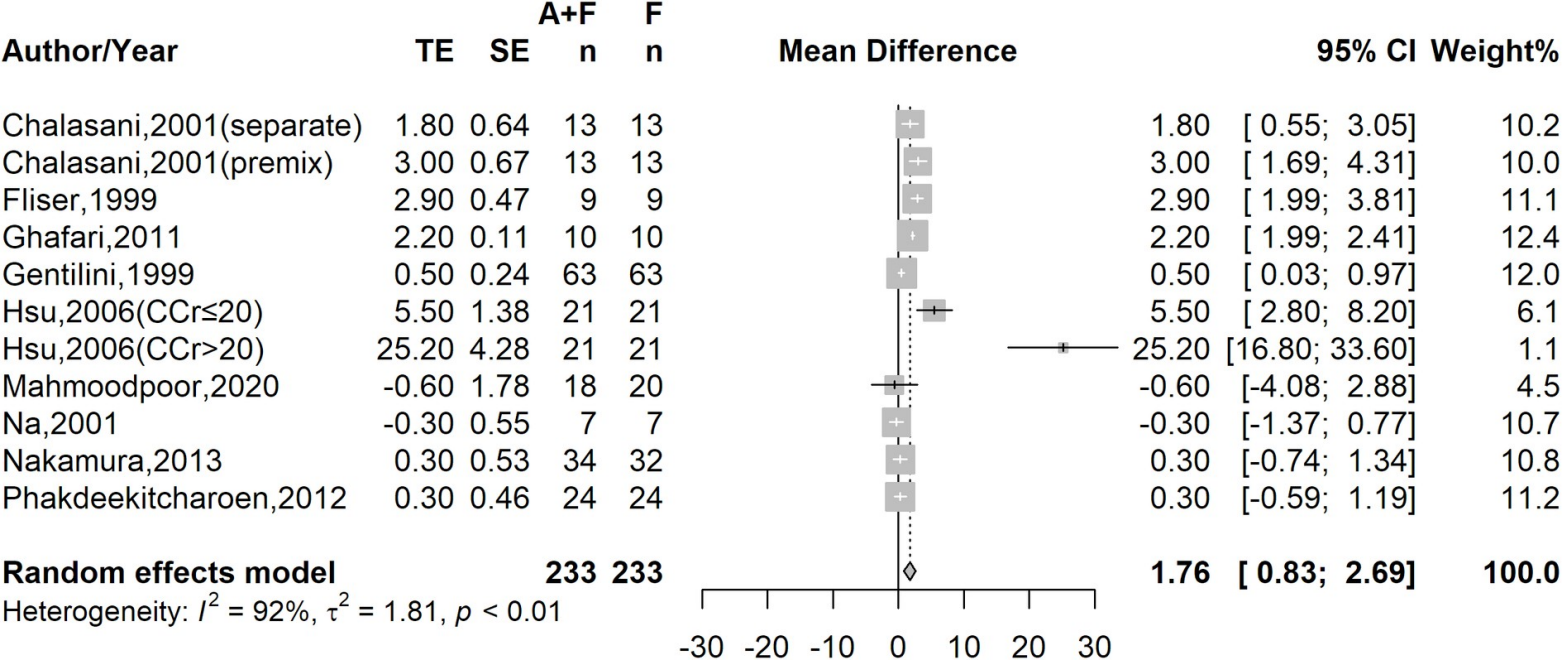
Treatment effect of co-administration furosemide with albumin on urinary sodium excretion rate.

Five of the included studies reported urine sodium excretion within 6 to 8 hours after the co-administration of furosemide with albumin or the administration of furosemide alone, and 6 studies reported urine sodium excretion within 24 hours after treatment. In order to compare the treatment effect between the combination therapy and furosemide alone, we also unified the reported urinary sodium excretion rates. The meta-analysis showed that the co-administration of furosemide with albumin increased the mean urinary sodium excretion rate to 1.76 mEq/hour (95% CI, 0.83–2.69) above that with furosemide treatment alone (**Fig 2**). However, high heterogeneity across studies was detected (*I*^*2*^ = 92%, p<0.01).

### Sensitivity analysis and subgroup analysis

**Fig 3:**
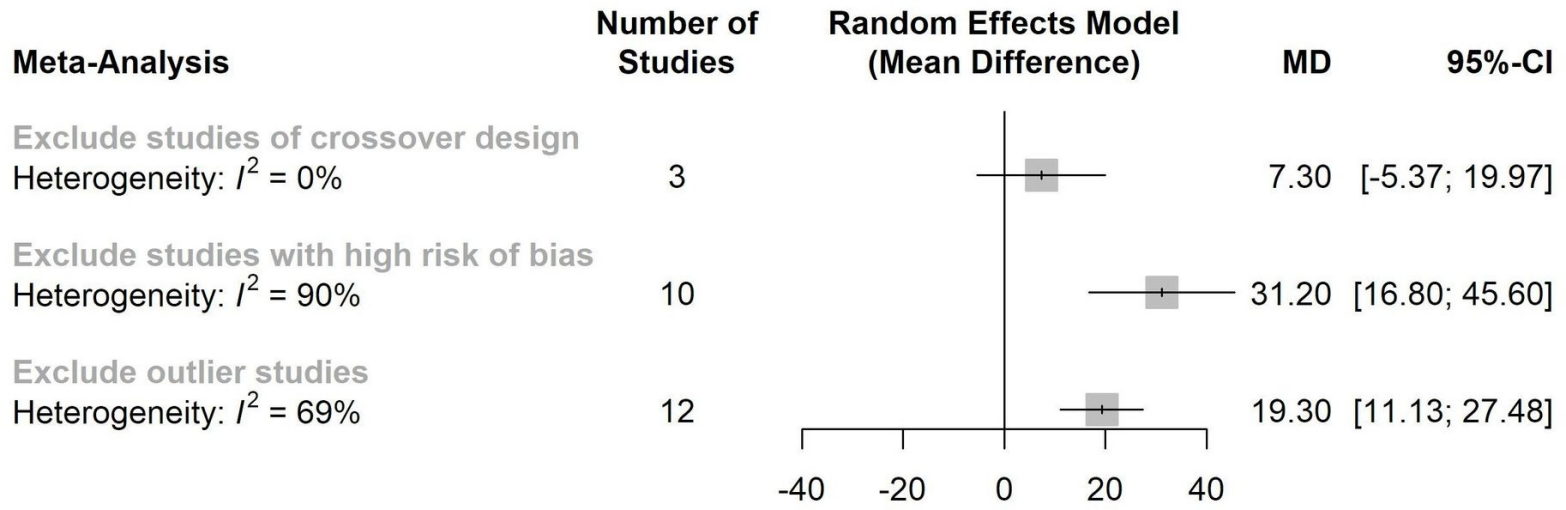
Sensitivity analysis of diuretic effect from co-administration furosemide with albumin.

**Fig 4:**
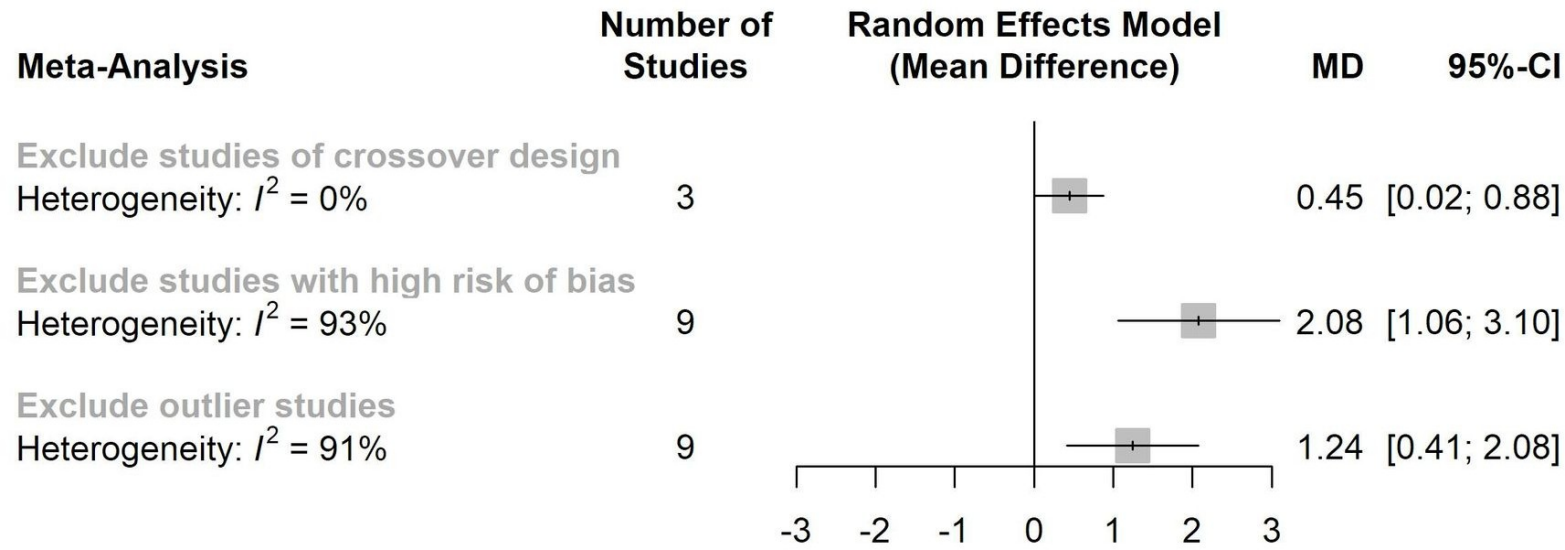
Sensitivity analysis of natriuretic from co-administration furosemide with albumin.

In considering the urine output rate as an outcome, a sensitivity analysis was conducted to assess the robustness of the results. After removing the data from an outlier study (Hsu, 2006) [28], the statistical heterogeneity was reduced from 87% to 69%, and the combination therapy still had a significant effect in terms of increasing patients’ urine output (**Fig 3**). Further analysis established that the combination therapy maintained its advantage even after the studies with high risk of bias were removed. Interestingly, after excluding the studies with crossover designs, only three studies were included in the subsequent sensitivity analysis, and that analysis revealed only a trend toward improved urine output rates from the combination therapy in comparison with furosemide monotherapy. Another sensitivity analysis was performed to assess the robustness of the results regarding the natriuretic effect of albumin and furosemide co-administration. There was still benefit from albumin and furosemide co-administration after excluding the studies with crossover designs and excluding outlier studies or studies with high risk of bias (**Fig 4**).

**Fig 5:**
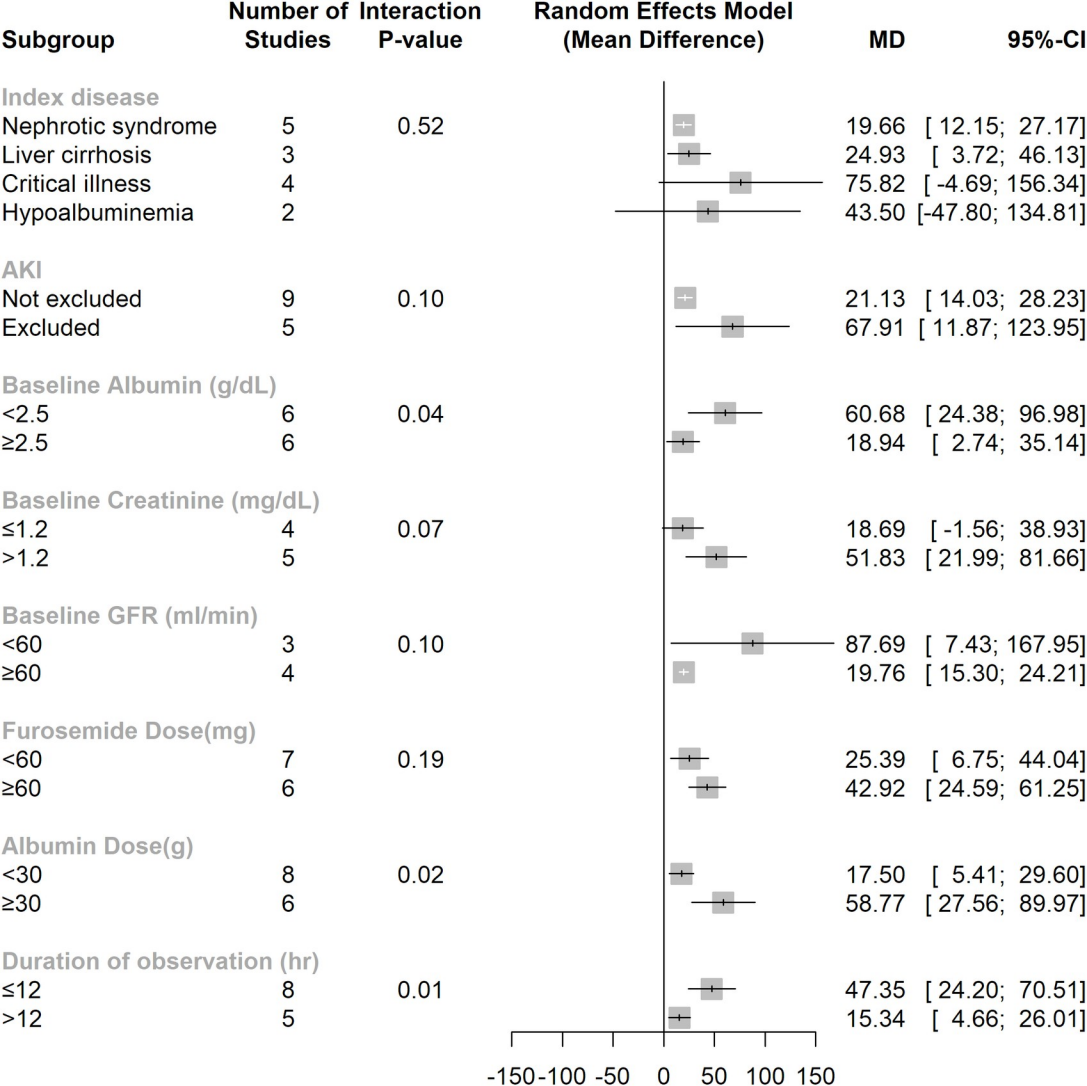
Subgroup analysis of diuretic effect from co-administration furosemide with albumin.

We next conducted subgroup analyses to explore potential treatment effect heterogeneity. From the subgroup analysis of urine output rates, we found that the index disease, when excluding acute kidney injury (AKI) populations and furosemide dose (≥ 60 mg or < 60 mg), did not result in significant treatment effect differences. Further subgroup analyses revealed significant modification effects for four subgroup variables: baseline albumin level (≥ 2.5 g/dL or < 2.5 g/dL) (interaction P value = 0.04), baseline creatinine level (> 1.2 mg/dL or ≤ 1.2 mg/dL) (interaction P value = 0.07), prescribed albumin dose (≥ 30 g or < 30 g) (interaction P value = 0.02), and duration of observation (≤ 12 hours or > 12 hours) (interaction P value = 0.01) (**Fig 5**). Trend of better diuretics effect was observed in those with eGFR less than 60 ml/min/1.73m2 but without statistical significance (interaction P value = 0.1).

**Fig 6:**
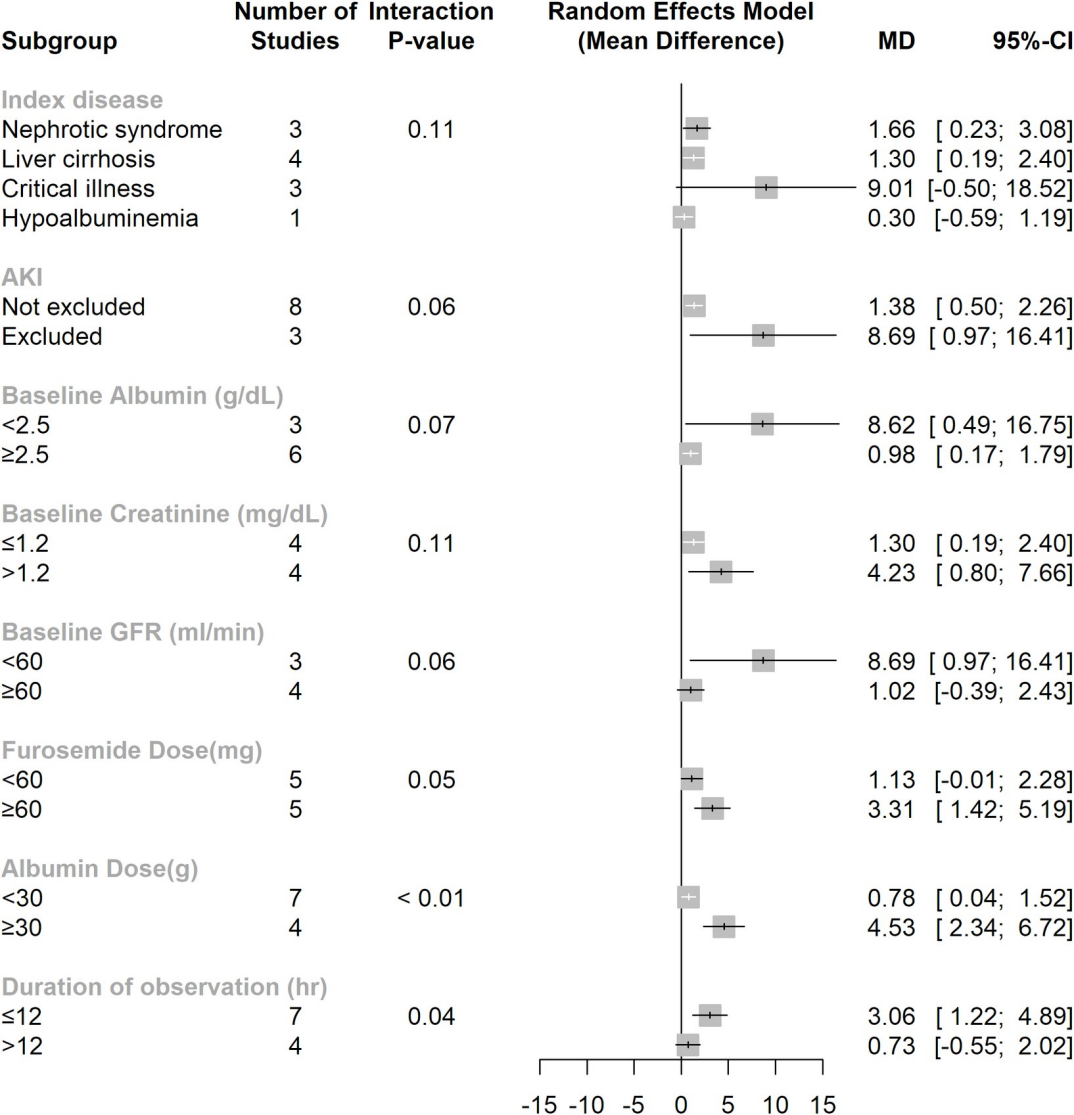
Subgroup analysis of natriuretic from co-administration furosemide with albumin.

No significant modification effect was detected regarding the natriuretic effect of albumin and furosemide co-administration for two subgroup variables: index disease and baseline creatinine level. The subgroup analyses further revealed a significant modification effect on urine sodium excretion for five subgroup variables: baseline albumin level (≥ 2.5 g/dL or < 2.5 g/dL) (interaction P value = 0.07), prescribed furosemide dose (≥ 60 mg or < 60 mg) (interaction P value = 0.05), prescribed albumin dose (≥ 30 g or < 30 g) (interaction P value < 0.01), and duration of observation (≤ 12 hours or > 12 hours) (interaction P value = 0.04), and baseline eGFR (≥ 60 ml/min/1.73m2 or < 60 ml/min/1.73m2) (interaction P value = 0.06). Studies excluding AKI patients also demonstrated a better natriuretic effect from the co-administration of albumin and furosemide in comparison to studies not excluding AKI patients (interaction P value = 0.06) (**Fig 6**). Overall, the subgroup analyses demonstrated that lower baseline albumin levels and higher prescribed albumin doses were both associated with better treatment effects from the co-administration furosemide with albumin in terms of urine output and urine sodium excretion.

### Assessment of evidence quality and summary of findings

We evaluated the primary outcome and performed quality assessment using the GRADE system. The outcomes and assessments are presented as a summary of findings in **S3 Table**.

## Discussion

In the present meta-analysis, a total of 13 studies with a total of 422 participants were included, and three points are worth summarizing. First, the co-administration of furosemide with albumin increased the urine output by 31.45ml/hour and increased the urine sodium excretion rate by 1.76 mEq/hour in comparison to furosemide treatment alone. Second, the diuretic and natriuretic effects of albumin and furosemide co-administration were better in participants with low baseline serum albumin levels (< 2.5 g/dL) and high prescribed albumin infusion doses (> 30 g). Third, the potentiation of diuresis and natriuresis from the combination of albumin and furosemide might have been more prominent within the first 12 hours after administration. Fourth, the diuretic or natriuretic effect from co-administration might be better in those with baseline impaired renal function (eGFR < 60 ml/min/1.73m2 or creatinine 1.2 mg/dL).

By including 13 studies in the present meta-analysis, it was found that the co-administration of furosemide with albumin resulted in statistically significant increases in urine output and urine sodium excretion rates than furosemide monotherapy. Although the present meta-analysis revealed that co-administration therapy increased the urine output by 31.45ml/hour, its efficacy in resolving edema is still indefinite. Within these enrolled studies, only one study reported higher body weight reduction from co-administration therapy in comparison to the furosemide therapy alone. Martin and his colleagues had revealed that co-administration therapy can provide body weight reduction 2.2kg in the first 24 hours and 7.4kg in 72 hours and the furosemide therapy provides body weight reduction 2.2kg in the first 24 hours and 5.4kg in 72 hours.

Through subgroup analysis, the present study demonstrated that the co-administration of furosemide and albumin showed significantly greater diuretic and natriuretic effects in patients with baseline albumin levels lower than 2.5 g/dL. Aside from the baseline albumin level, the dose of albumin prescribed also made a difference in the natriuretic and diuretic effects observed, which were significantly increased when the dose of albumin prescribed was more than 30 g. The influence of these two factors, baseline albumin level and dose of treatment, failed to be demonstrated in previous meta-analyses [11]. Pichette et al. had reported that hypoalbuminemia is associated with an increase in the renal metabolic clearance of furosemide, possibly because of the increase in the concentration of unbound furosemide. This increased renal metabolic clearance of furosemide could lead to a reduction in active form furosemide tubular secretion in the S1 segments of proximal tubules [6,32]. Later studies further confirmed that albumin infusion in hypoalbuminemic patients does increase renal furosemide excretion [21]. In 2019, Ellison pointed out that the failure of some studies to support the co-administration strategy might have been due to the relatively high mean serum albumin levels in hypoalbuminemia groups, such as the mean albumin level of 3.4 g/dL in Charokopos’ trial and the mean albumin level of 3.0 g/dL in Chalasani’s trial, in comparison to those in animal models. He concluded that patients with hypoalbuminemia > 2.0 g/dL are unlikely to benefit from albumin infusion with furosemide treatment [25,33], while patients with serum albumin levels < 2.0 g/dL could potentially benefit from the co-administration of furosemide with albumin [5,21]. According to the previous reports and present study, the degree of hypoalbuminemia seems to influence the efficacy of co-administration therapy, but it’s noteworthy that the diuretics resistance might be related to decreasing kidney function, use of nonsteroidal anti-inflammatory drugs (NSAIDs), congestive heart failure or poor salt restriction [1–3]. These possible factors leading to diuretics resistance should be evaluated and minimized before prescription co-administration therapy.

We observed potential better diuretics or natriuresis effects of the co-administration of furosemide and albumin in those with impaired renal function (identified by eGFR less than 60 ml/min/1.73m2 or creatinine 1.2 mg/dL). The creatinine clearance was the major determinant of furosemide diuretic efficiency. Chronic kidney disease and heart failure all can result in a right shift of the relationship between sodium excretion rate and renal tubular furosemide secretion rate [3,34]. Higher threshold point is also noted in those with impaired renal function. Further, furosemide is transported to the active site via organic anion transporters, increasing plasma levels of organic anion (OA) in chronic kidney disease (CKD) that compete with the peritubular uptake of furosemide while metabolic acidosis depolarizes the membrane potential of proximal tubule cells which further decrease OA secretion in CKD [35–37]. As above mentioned, co-administration of albumin and furosemide might increase the secretion of active form furosemide, which might be the explanation of better diuretic effect of this co-administration.

The subgroup analyses in the present study also revealed that the urine output rate and urine sodium excretion rate were significantly increased in the first 12 hours after the co-administration of furosemide and albumin. Similar findings were also noted in a previous meta-analysis, in which Kitsios and his colleague concluded that the increase in urine volume due to the combination of furosemide and albumin was only statistically significant at 8 hours but no longer significant at 24 hours after administration [11]. According to previous pharmacokinetic and pharmacodynamic data, the plasma elimination half-life of intravenous infusion furosemide is approximately 0.6 hours, and it is mainly eliminated via the kidneys [37]. The treatment effect of furosemide with albumin seems to last for 8 to 12 hours after administration, so it is reasonable to prescribe the co-administration of furosemide and albumin at the frequency of every 12 hours to maintain the diuretic and natriuretic effects. Considering the relatively short half-life and treatment effect of furosemide and albumin co-administration therapy and furosemide alone, it might be more suitable for inpatient treatment instead of outpatient treatment strategy.

### Strengths and limitations of this study

The strength of our meta-analysis included the following: (1) The present study did clarify the benefit of furosemide and albumin co-administration in terms of diuresis and natriuresis. (2) Considering the heterogeneity of index diseases and the diversity in baseline characteristics and doses of the treatment, subgroup analyses were performed in our study. Through the subgroup analyses, this study revealed statistically significant benefits of furosemide and albumin co-administration in patients with hypoalbuminemia lower than 2.5 mg/dL or those receiving albumin doses of more than 30 g. These findings, which did not discussed in previous meta-analysis [11], give the clinical physician more confidence in choosing appropriate patients and treatment dosages when utilizing furosemide and albumin co-administration. (3) We also conducted a sensitivity analysis to examine the results after excluding the crossover trials.

The limitations of our review included the following: (1) The total number of patients included was still limited, and there was high statistical heterogeneity among the included trials, including in terms of the enrolled populations, their underlying characteristics, and treatment regimens. (2) There was diversity in the doses and types of albumin supplement and furosemide treatment used, and most of the studies did not standardize their pharmacologic treatment according to patient body weight. (3) Several of the included studies had crossover designs, and some of them did not provide the average albumin level after the washout phase and before each intervention. Thus, we could not confirm that the serum albumin level changed in those patients who received furosemide and albumin co-administration before receiving furosemide treatment alone. (4) Data on other factors that might have influenced the diuretic effects were not recorded in some of the studies, including data on salt restriction strategies used, NSAID prescriptions, and probenecid prescriptions. (5) The furosemide dose varies across studies. We choose 60mg furosemide as the cutoff point for subgroup analysis, which could make the number of enrolled studies split equally for analyses. We also choose this stress dose furosemide (1mg furosemide per kilogram body weight) as the cutoff point for evaluating the tubular function under the assumption of the enrolled population with an average 60 kg body weight. However, the furosemide dose-response in patients with hypoalbuminemia and the diuretic response when in combination with albumin warranted further examining. (6) Most of the enrolled studies were cross-over designs and sensitivity analysis was performed to exclude cross-over design trials with the remaining only 3 enrolled trials with parallel design. Further well-designed prospective trials or analyses were needed to explore the source of treatment effect heterogeneity.

## Conclusion

Co-administration of furosemide with albumin might enhance diuresis and natriuresis effects than furosemide treatment alone but with high heterogeneity in treatment response. According to the present meta-analysis result, combination therapy might provide advantages compared to the furosemide therapy alone in patients with baseline albumin levels lower than 2.5 g/dL. Owing to high heterogeneity and limited enrolled participants, further parallel randomized controlled trials are warranted to examine our outcome.

## Supporting information

S1 Checklist PRISMA 2009 checklist. (DOC)

S1 Fig PRISMA flow chart of study inclusion. (TIF)

S2 Fig Risk of bias assessment of each studies. (TIF)

S3 Fig Summary of risk of bias. (TIF)

S4 Fig Funnel plot for evaluation of publication bias of urine output rate. (TIF)

S5 Fig Funnel plot for evaluation of publication bias of urinary sodium excretion rate. (TIF)

S1 Table Details of Search Strategy results from source: Pubmed (A), EMbase (B) and from Medline (C).
(DOCX)

S2 Table GRADE evidence and summary of findings table. (DOCX)

S3 Table Primary reasons for exclusion of excluded studies after full text reviewed. (DOCX)

## Abbreviations

AKI: acute kidney injury
CKD: chronic kidney disease
NSAID: nonsteroidal anti-inflammatory drugs
OA: organic anion
SD: standard deviations
