# Supplementary figures and images for: Diuretic effect of co-administration of furosemide and albumin in comparison to furosemide therapy alone: An updated systematic review and meta-analysis
Source: PLoS One. 2021 Dec 1;16(12):e0260312. doi: 10.1371/journal.pone.0260312 (PMC8635380; doi:10.1371/journal.pone.0260312)

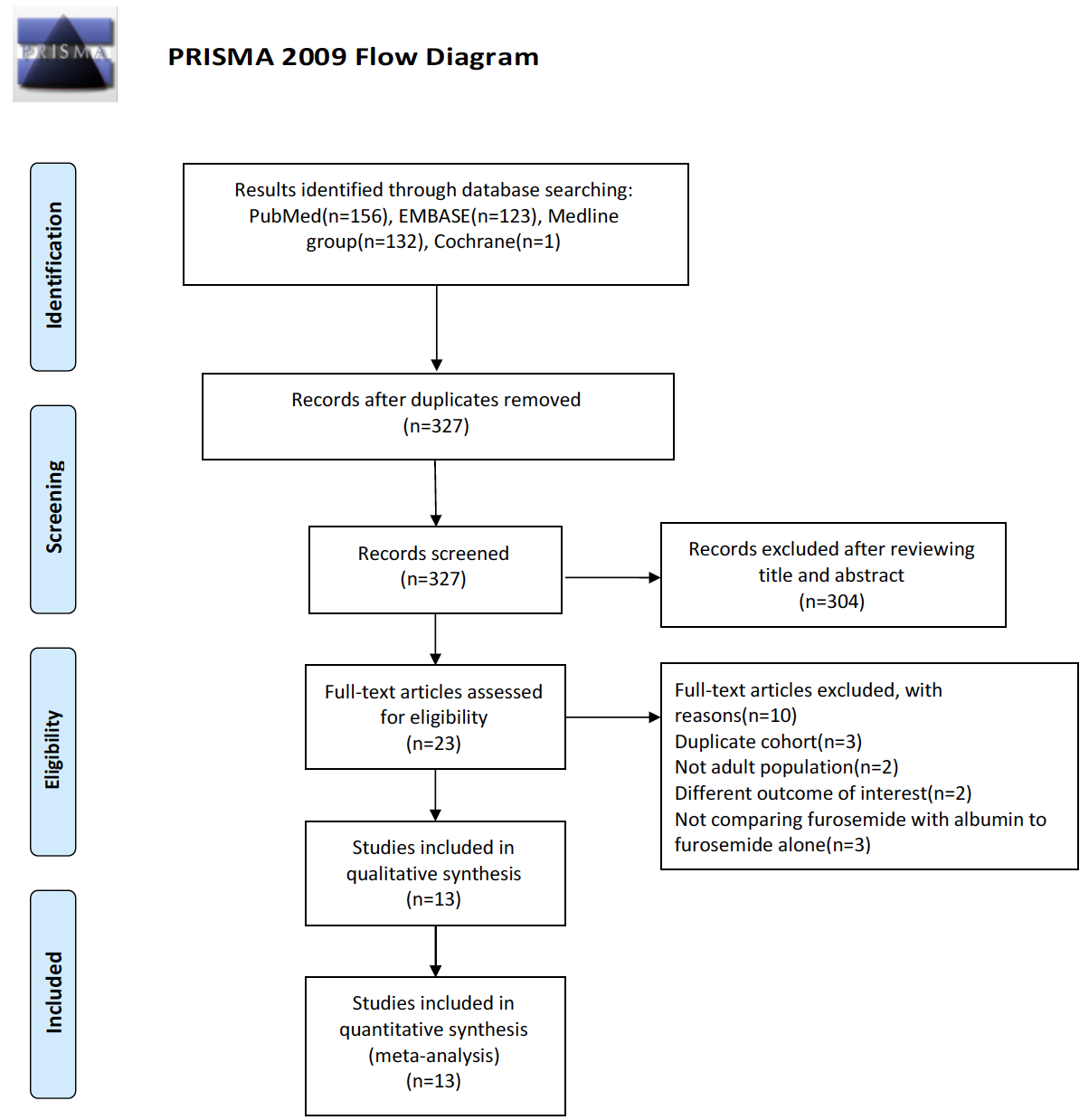

Supplement: S1 Fig — (TIF) [file pone.0260312.s002.tif]

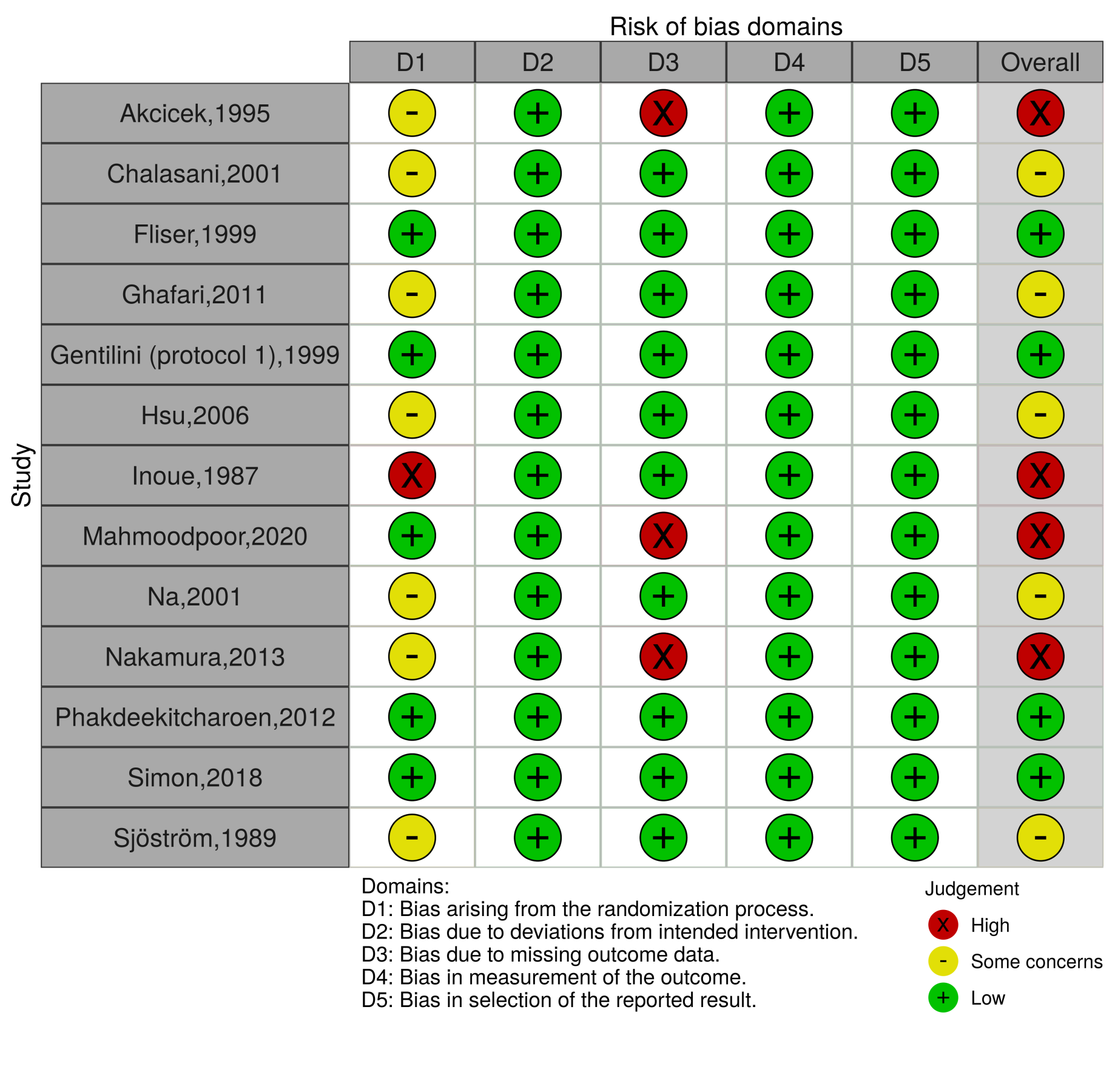

Supplement: S2 Fig — (TIF) [file pone.0260312.s003.tif]

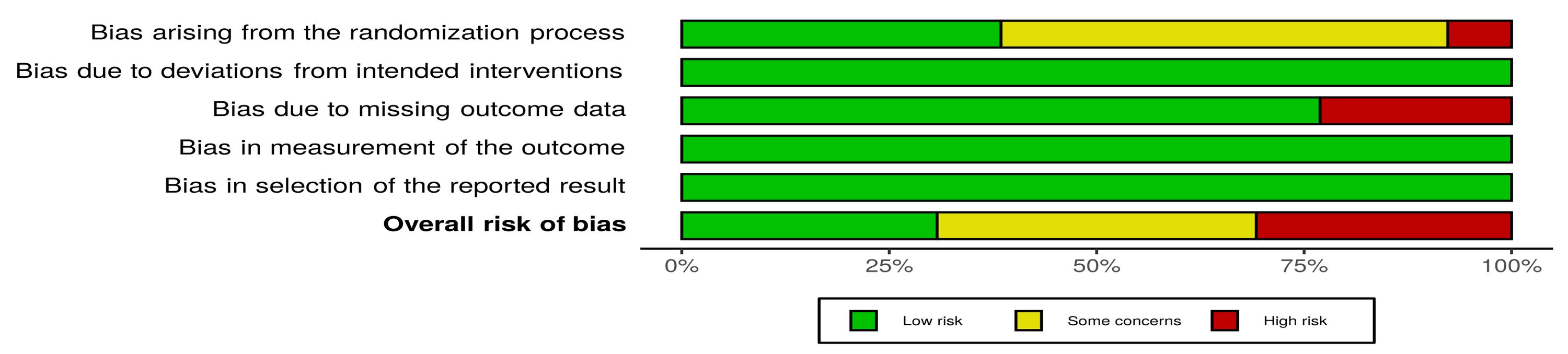

Supplement: S3 Fig — (TIF) [file pone.0260312.s004.tif]

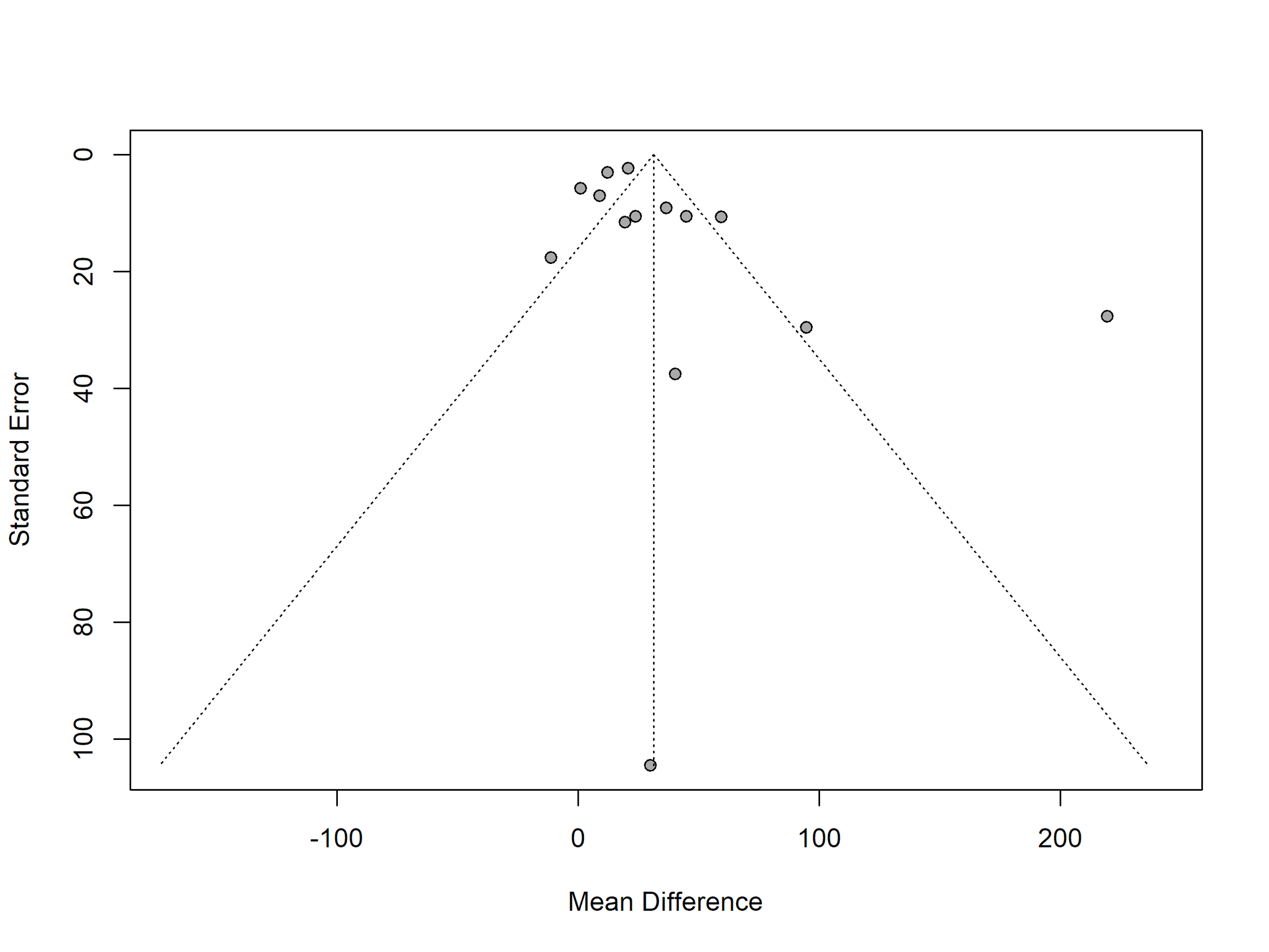

Supplement: S4 Fig — (TIF) [file pone.0260312.s005.tif]

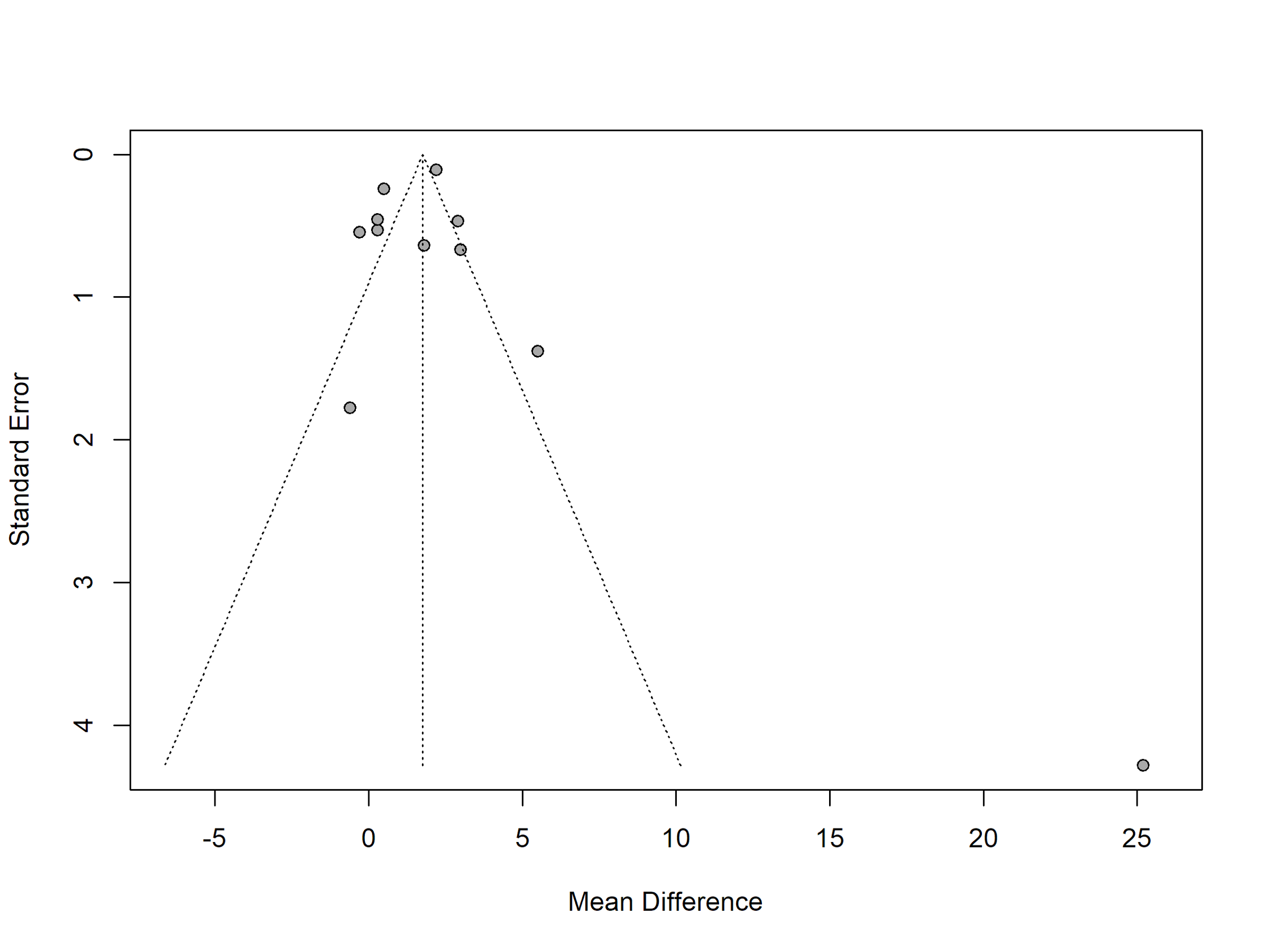

Supplement: S5 Fig — (TIF) [file pone.0260312.s006.tif]
