# Supplementary material for: Diuretic effect of co-administration of furosemide and albumin in comparison to furosemide therapy alone: An updated systematic review and meta-analysis
Source: PLoS One. 2021 Dec 1;16(12):e0260312. doi: 10.1371/journal.pone.0260312 (PMC8635380; doi:10.1371/journal.pone.0260312)
Supplement: S1 Table — Details of Search Strategy results from source: Pubmed (A), EMbase (B) and from Medline (C). (DOCX) [file pone.0260312.s007.docx]

**Supplementary Table 1A Details of Search Strategy Source: Pubmed; Searched on: 18,Oct, 2020;**

| Search | Query | Items |
| --- | --- | --- |
| [#1](https://www.ncbi.nlm.nih.gov/pubmed/advanced) | "furosemide"[Mesh] | 11879 |
| #2 | "lasix"[Title/Abstract] | 339 |
| #3 | "furosemide"[Title/Abstract] | 11959 |
| #4 | “diuretics” [Mesh] | 35372 |
| #5 | “diuretics” [Title/Abstract] | 23366 |
| #6 | [#1](https://www.ncbi.nlm.nih.gov/pubmed/advanced) OR [#2](https://www.ncbi.nlm.nih.gov/pubmed/advanced) OR #3 OR 4# OR 5# | 59008 |
| #7 | " hypoalbuminemia "[Mesh] | 1236 |
| #8 | " hypoalbuminemia "[Title/Abstract] | 5404 |
| #9 | "Albumin"[Title/Abstract] | 153042 |
| #10 | "Albumins"[Mesh] | 176307 |
| #11 | #7 OR #8 OR #9 OR #10 | 267981 |
| #12 | #6 AND #11 | 1442 |
| #13 | #12 + Filter: RCT | 156 |

**Supplementary Table 1B Details of Search Strategy Source: Embase; Searched on: 18,Oct, 2020**

| **Search** | **Query** | **Items** |
| --- | --- | --- |
| [#1](https://www.ncbi.nlm.nih.gov/pubmed/advanced) | furosemide:ab,ti OR loop diuretics:ab,ti | 68371 |
| #2 | 'albumin':ab,ti OR 'hypoalbuminemia':ab,ti | 290638 |
| #3 | [#1](https://www.ncbi.nlm.nih.gov/pubmed/advanced) AND [#2](https://www.ncbi.nlm.nih.gov/pubmed/advanced) | 3350 |
| #4 | #1 AND #2 AND [humans]/lim AND randomized controlled trial/lim | 123 |

**Supplementary Table 1C Details of Search Strategy Source: Medline; Searched on: 18,Oct, 2020**

| **Search** | **Query** | **Items** |
| --- | --- | --- |
| [#1](https://www.ncbi.nlm.nih.gov/pubmed/advanced) | (Albumin or hypoalbuminemia).mp. | 187665 |
| #2 | (furosemide or loop diuretics).mp. | 18299 |
| #3 | [**#1**](https://www.ncbi.nlm.nih.gov/pubmed/advanced) **AND** [**#2**](https://www.ncbi.nlm.nih.gov/pubmed/advanced) | 559 |
| #4 | **#1 AND #2 , Filter: Adult AND human** | 132 |
