# Supplementary material for: Diuretic effect of co-administration of furosemide and albumin in comparison to furosemide therapy alone: An updated systematic review and meta-analysis
Source: PLoS One. 2021 Dec 1;16(12):e0260312. doi: 10.1371/journal.pone.0260312 (PMC8635380; doi:10.1371/journal.pone.0260312)
Supplement: S2 Table — (DOCX) [file pone.0260312.s008.docx]

**Supplemental Table 2 GRADE Evidence and Summary of Findings Table**

| Certainty assessment | | | | | | | № of patients | | Effect | | Certainty | Importance |
| --- | --- | --- | --- | --- | --- | --- | --- | --- | --- | --- | --- | --- |
| № of studies | Study design | Risk of bias | Inconsistency | Indirectness | Imprecision | Other considerations | co-administration of albumin and furosemide | furosemide | Relative (95% CI) | Absolute (95% CI) |  |  |
| Diuretics effect (assessed with: urinary output rate (ml/hr)) | | | | | | | | | | | | |
| **12** | **randomised trials** | **serious^a^** | **serious^b^** | **not serious** | **serious^c^** | **dose response gradient** | **223** | **220** | **-** | **MD** 31.45 ml/hour higher **(19.3 higher to 43.59 higher)** | **⨁⨁◯◯ Low** | **IMPORTANT** |

CI: confidence interval; MD: mean difference

**Explanations**

a. Four of enrolled studies have high overall risk of bias (Akcicek,1995; Inoue,1987; Mahmoodpoor,2020; Nakamura,2013) according to RoB2 assessment

b. Potential inconsistency was detected between subgroups and studies with different design

c. The total sample size is not large and the mean difference is relative small
