# Supplementary material for: Diuretic effect of co-administration of furosemide and albumin in comparison to furosemide therapy alone: An updated systematic review and meta-analysis
Source: PLoS One. 2021 Dec 1;16(12):e0260312. doi: 10.1371/journal.pone.0260312 (PMC8635380; doi:10.1371/journal.pone.0260312)
Supplement: S3 Table — (DOCX) [file pone.0260312.s009.docx]

**Supplementary Table 3** Primary reasons for exclusion of excluded studies after full text reviewed

| First Author | **Last Author** | **Journal** | **Year** | **Title** | **Primary reason for exclusion** | **Include** |
| --- | --- | --- | --- | --- | --- | --- |
| Ali Ghafari1 | Nariman Sepehrvand | Saudi Journal of Kidney Diseases and Transplantation | 2011 | Co-administration of Albumin–Furosemide in Patients with the Nephrotic Syndrome | Duplication cohort | N |
| Asjad Khan | Ramesh Venkataraman | Critical Care | 2007 | Albumin and furosemide for acute lung injury | Duplication cohort | N |
| D.Craig Brater | Juan Maya | Transaction of the American Clinical and  Climatological Association | 2001 | Effect of albumin-furosemide mixtures on response to furosemide in cirrhotic patients with ascites | Duplication cohort | N |
| Greg S. Martin | Gordon R. Bernard | Critical Care Medicine | 2002 | Albumin and furosemide therapy in hypoproteinemic patients with acute lung injury | Comparing treatment effect of furosemide with albumin to placebo | N |
| Greg S. Martin | Gordon R. Bernard | Critical Care Medicine | 2005 | A randomized, controlled trial of furosemide with or without albumin in hypoproteinemic patients with acute lung injury | Different outcome of interest | N |
| Rajmohan Dharmaraj | Arvind Bagga | Pediatric Nephrology | 2009 | Randomized cross-over trial comparing albumin and furosemide infusions in nephrotic syndrome | Not adult population | N |
| Rahul Pathak | Prem Khadga | Journal of Nepal Health Research Council | 2020 | Comparative Study of Slow Infusion versus Bolus Doses of Albumin and Furosemide Infusion to Mobilize Refractory Ascites in Decompensated Chronic Liver Disease | Comparing treatment effect of bolus or continuous fusion furosemide with albumin | N |
| Reddy, M. | Bansal, A. | Pediatric Critical Care Medicine | 2018 | Abstract O-46: albumin and furosemide for diuresis in critically ill children with fluid overload-a pilot randomized double blind placebo controlled trial | Not adult population | N |
| Toru Nakamura | Michio Imawari | Molecular and Clinical Oncology | 2013 | Contribution of diuretic therapy with human serum albumin to the management of ascites in patients with advanced liver cirrhosis: A prospective cohort study | Different outcome of interest | N |
| Xing Chen | Zi Hong Ma | Hunan Yi Ke Da Xue Xue Bao | 2001 | Effect of dextran combination with furosemide on nephrotic syndrome | Comparing treatment effect of furosemide with dextran and furosemide with albumin | N |
